# Mentalisierungsfähigkeit und Empathie in der Psychiatrie – eine Sozialisationsfrage?

**DOI:** 10.1007/s00739-021-00738-9

**Published:** 2021-07-31

**Authors:** D. Steinmair, B. Fink, E. Horvath, K. Matuszak-Luss, H. Löffler-Stastka

**Affiliations:** 1 Klinik für Psychoanalyse und Psychotherapie, Medizinische Universität Wien, Währinger Gürtel 18–20, 1090 Wien, Österreich; 2 Klinische Abteilung für Augenheilkunde und Orbitachirurgie, Universitätsklinikum St. Pölten, Karl Landsteiner Privatuniversität für Gesundheitswissenschaften, St. Pölten, Österreich; 3 Sektion Psychotherapie/AG Ambulante Psychotherapie, Österreichische Gesellschaft für Psychiatrie, Psychotherapie und Psychosomatik, Wien , Österreich; 4 Ordination, Auhofstraße 70/4, 1130 Wien, Österreich; 5 Ordination, Tellgasse 14/12, 1150 Wien, Österreich; 6 Ordination für Psychiatrie, psychotherapeutische Medizin und Neurologie, Seckendorfstraße 6/1/6, 1140 Wien, Österreich

**Keywords:** Identität, Innere Objekte, Sekundäre/Berufliche Sozialisation, Resilienz, Reflexion, Identity, Internal objects, Secondary socialization, Resilience, Reflective functioning

## Abstract

Berufliche Sozialisation entwickelt sich im Kontext der Persönlichkeitsentwicklung und vorhandener Bedingungen unterschiedlich. Das Aufrechterhalten der Mentalisierungsfähigkeit im beruflichen Alltag und vor allem in herausfordernden Situationen ist keine Selbstverständlichkeit, sondern abhängig von Umgebungsbedingungen. Die Übernahme von Verantwortung für die gewählte berufliche Tätigkeit erfordert eine Auseinandersetzung mit dem jeweils spezifischen Anforderungsprofil; es stellen sich Lern- und Entwicklungsaufgaben. Die Integration der beruflichen Identität im Rahmen förderlicher Umgebungsbedingungen bedeutet, sich als Teil dieser Gruppe(n) zu erleben. Aus gruppenpsychoanalytischer Sicht stellt die Identifikation mit einer Gruppe eine Verinnerlichung eines „guten Objekts“ dar, einer Gruppenrepräsentanz, welche in herausfordernden Situationen zugänglich ist. Dies kann die Mentalisierungsfähigkeit verbessern.

## Abstract (en)

Professional socialization develops differently in the context of personality development and existing conditions. Preserving mentalization in everyday professional life, and especially in challenging situations, is not taken for granted but depends on the context. The assumption of responsibility when choosing a profession requires an examination of the specific occupational profile in each case; learning and development tasks arise. The integration of professional identity in favourable circumstances means feeling like a part of this/these group(s). From a group-analytical viewpoint, identification with a group represents internalization of a “good object,” a group representation that is accessible in challenging situations. This may improve mentalization.

## Mentalisierungsfähigkeit

Die Entwicklung der Mentalisierungsfähigkeit beginnt in den ersten Lebensmonaten. Mentalisierung ist eine in sozialer Interaktion auf der Basis einer sicheren Bindungsbeziehung erworbene Fähigkeit, eine Kompetenz, welche wiederum Voraussetzung für eine erfolgreiche Interaktion ist. Sie beruht darauf, Gedanken und Gefühle bei sich selbst und in anderen vorauszusetzen und dieses vermutete Innenleben mit der Realität zu verknüpfen [1]. Mentalisierungsfähigkeit ist mit Empathie und reflexiver Kompetenz verbunden [2]. Konzeptualisierungen von Empathie integrieren eine affektive reflektorische Komponente mit kognitiven und sozialen Aspekten. Empathie beinhaltet die reflektorische Nachahmung (Gefühlsansteckung, Projektion; vgl. [3, 4]), ein Sichhineinfühlen [5], aber auch die Fähigkeit der Perspektivenübernahme [6–8]. Empathie ist verknüpft mit sozialen Interessen und Wertvorstellungen [9–13].

Die Entwicklung eines integrierten Selbstkonzepts, das Finden einer Identität sind Aufgaben, welche im adoleszenten Alter zu bewältigen sind. Reflexive Kompetenz und Mentalisierungsfähigkeit sind Voraussetzungen für das Sichablösen von Bezugspersonen und die Nachahmung von Rollenstereotypen – wobei in der Adoleszenz eine Abhängigkeit von der Anerkennung anderer besteht.

## Berufliche Sozialisation

Sozialisation ist ein Prozess, welcher über die Aneignung von Wissen hinausgeht. Im Laufe dieser Sozialisation kommt es zur Formung einer beruflichen Identität; eigene Haltungen und Werte sowie Interaktionsroutinen und Verhaltensweisen werden mit jenen abgestimmt, welche in der Gemeinschaft vermittelt werden [14, 15].

Als Einflüsse auf die Sozialisation wurden neben Voraussetzungen und bestehenden Organisationsstrukturen (z. B. Lernumgebung und Klima, Belohnungssysteme, Diversitätsmanagement) vor allem soziale Interaktionen identifiziert (z. B. mit Vorbildern und Mentoren, Fachkollegen und interdisziplinären Kontakten) (vgl. [14]). Narrative über Stereotype, Imitation von Rollenvorbildern, aber auch Gewöhnung (Routinen, soziale Normen) tragen zur Sozialisation bei [16].

Im Spannungsfeld von Vorschriften und Strukturen passiert eine Auseinandersetzung mit den vermittelten Erwartungen, Pflichten, Werten und Normen, welche mit der Ausformung einer beruflichen Identität einhergehen. Diese Entwicklung basiert auf Erfahrungen, der Auseinandersetzung mit Rollenvorbildern und auf Reflexion.

## Interprofessionelle Teams

Interprofessionelle Teams stehen vor der Herausforderung der Zusammenarbeit zwischen verschiedentlich spezialisierten und sozialisierten Berufsgruppen. Eine gute Kooperation benötigt Maßnahmen zum Teambuilding, Anerkennung der jeweiligen Expertise und Förderung von Strukturen, welche die Zusammenarbeit fördern (z. B. gemeinsame Fort- und Weiterbildung; vgl. auch [17]).

## Psychische Erkrankungen in Österreich, Versorgungssituation

Die psychotherapeutische Versorgung von Menschen mit psychischen Symptomen erfolgt in Österreich vor allem durch Psychotherapeuten (*N* = 10.415) als auch durch Fachärzte für Psychiatrie, etwa die Hälfte mit psychotherapeutischer Medizin als Zusatzqualifikation, und in der allgemeinmedizinischen Versorgung [18]. Interdisziplinarität beinhaltet in der Psychiatrie einen Austausch zwischen verschiedenen Berufsgruppen, die Versorgungsbereiche im (psycho)pharmakologischen, psychotherapeutischen und sozialen Spektrum umfassen.

Die Prävalenz psychischer Krankheiten in der allgemeinen Bevölkerung (18- bis 65-jährig, österreichweit) bezogen auf ein Jahr betrug 22,7 % [19]. Nur ein kleiner Teil der von einer psychischen Erkrankung Betroffenen erhält eine Behandlung [19]. Es besteht in 86 % eine Komorbidität mit einer körperlichen Erkrankung [19]; dies hat Implikationen auf die Planung von Versorgung.

## Mentalisierungsfähigkeit und Sozialisation

Eine gute Mentalisierungsfähigkeit wirkt protektiv in stressigen Situationen und geht mit erhöhtem Wohlbefinden einher (vgl. [20]). Welche Mediatoren und Moderatorvariablen beeinflussen die Mentalisierungsfähigkeit?

In Laverdières Analyse von Empathie in „mental health professionals“ mit unterschiedlicher Berufserfahrung zeigten sich unterschiedliche Profile (vgl. [21]). Die Berufserfahrung wurde neben dem Geschlecht des Therapeuten, beruflichem Hintergrund und theoretischer Spezialisierung als Faktor mit Auswirkung auf die Empathie identifiziert. Die Fähigkeit zum Perspektivenwechsel nahm zu, während Fantasiefähigkeit, Betroffenheit und Distress abnahmen (vgl. Interpersonal Reactivity Index [21, 22]). Durch Berufserfahrung ist Distanz zum Einzelfall gegeben, mögliche und notwendige therapeutische Interventionen können aus dieser Position antizipiert werden. Eine Einordung des Falls und Kontexts wird aufgrund der Erfahrung und des Wissens ermöglicht. Distress, emotionale Betroffenheit in der Begegnung oder eine Identifizierung wird vermindert; eine bessere Abgrenzung zwischen Selbst und Gegenüber macht Gegenübertragungs- und Übertragungsphänomene therapeutisch nutzbar.

Metaanalysen sprechen klar für bessere Behandlungserfolge, wenn Interventionen mit Mitgefühl erfolgen [23]. Aus der WHO-Definition von Gesundheit, welche über das Fehlen von Krankheit hinausgeht, lässt sich schließen, dass diese Konzeption in die ärztliche Sozialisation einfließt [24]. Anders als die Empathiefähigkeit, welche mit zunehmender Berufserfahrung, wie oben beschrieben, zunimmt, verbessern sich kommunikative Fähigkeiten nicht durch Erfahrung [25], können aber trainiert werden. Kommunikationstraining für Angehörige verschiedenster Gesundheitsberufe zeigte eine signifikante Verbesserung dieser Skills [26]. Allerdings war der Effekt von Kommunikationstrainings auf die Zufriedenheit der behandelten Patienten nicht signifikant [26]. Für erfolgreiche Interventionen ist es nicht ausreichend, kommunikative Fähigkeiten zu beherrschen.

Einflüsse der Umgebungsbedingungen auf die Mentalisierungsfähigkeit sind sowohl während der Entwicklung dieser Fähigkeit [27] als auch im Erwachsenenalter beschrieben [28]. Die Mentalisierungsfähigkeit war bei Psychotherapeuten, welche im ambulanten Setting tätig sind, höher als bei Therapeuten im stationären Setting [28]. Unterschiedliche Arbeitsbedingungen und Unterschiede hinsichtlich der zu betreuenden Patienten wären mögliche Erklärungen.

Innerhalb der Berufsgruppen war in einer anderen Untersuchung [29] interessant, dass sich in einer Subgruppenanalyse ein (nicht signifikanter) Trend zeigte, dass die soziale Kognition bei verschiedenen Berufsgruppen innerhalb des stationären Settings unterschiedlich war (soziale Kognition gemessen mit MASC („Movie for the Assessment of Social Cognition“): PsychotherapeutInnenen/ErgotherapeutInnen/SozialarbeiterInnen bzw. NichtärztInnen > PsychiaterInnen > Pflegekräfte; MASC mean/(Range): 34,3/(25–40), 31,9/(24–41), 29,9/(18–39) [30]). Allerdings bedarf dieser beobachtete Trend einer Überprüfung in einer dafür ausgelegten Studie mit für diese Analyse ausreichender Stichprobe.

Empathie ist mit sozialen Interessen und Wertvorstellungen verknüpft

Untersuchungen des Einflusses von sozialem Status auf die Mentalisierungsfähigkeit fanden Unterschiede in der Art und Weise, wie die neuronale-kognitive Verarbeitung der sozialen Welt passiert, in Abhängigkeit vom sozialen Status [31].

Eine mögliche Erklärung wäre der Einfluss der Sozialisation, diese Hypothese müsste in entsprechenden randomisiert kontrollierten Studien (RCTs) überprüft werden. (Positive) Veränderung passiert eher in einer sozialen Umgebung, welche unterstützend ist, wenn sich das Gefühl der Selbstwirksamkeit entwickeln kann [32, S. 8].

Konstanz, Transparenz, klare Strukturen sowie klare Zuständigkeiten helfen im stationär psychiatrischen Setting, selbst in stressigen, affektiv aufgeladenen Situationen innezuhalten und sich Zeit zu nehmen für die Beziehungsarbeit, welche im Kontakt mit Patienten erforderlich ist.

## Fallvignette

Psychotherapeutische Gruppentherapien waren während der COVID-19-Pandemie aufgrund der Auflagen nicht durchgehend in Präsenz möglich. Gerade in der Behandlung von psychotischen Patienten, deren Beziehungserfahrungen von mangelnder Objektkonstanz und fehlenden verlässlich guten Objekten geprägt sind, ist das Etablieren und Aufrechterhalten eines stabilen Settings aber von zentraler Bedeutung.

Im Ambulatorium eines der Psychosozialen Dienste musste die fachärztlich geleitete gruppenanalytische Psychosentherapie, welche einmal wöchentlich kontinuierlich seit Jahren stattfand, unerwartet und zunächst mit unbestimmter Dauer unterbrochen werden. Auch, wenn die Gruppenleiterin während des ersten Lockdowns den Kontakt zu den Teilnehmern per Telefon/SMS hielt, war die Gruppe nicht präsent. Eine seit fünf Jahren teilnehmende Patientin wurde während dieser Zeit akut paranoid-psychotisch auf ihre Schwester, nachdem sie die Neuroleptika abgesetzt hatte, da sie sich „ja gut gefühlt“ hätte. Vor der Therapieunterbrechung waren ihre, zuvor über mehr als zehn Jahre fixierten, Wahnvorstellungen in den Hintergrund getreten. Dies hatte sich auch auf ihr Verhalten in der Gruppe auszuwirken begonnen, indem sie inzwischen ihren Stuhl nicht mehr aus dem Sesselkreis herausgerückt hatte, wie sie es zuvor über lange Zeit getan hatte. Im wiederaufgenommenen Therapieprozess konnte ihre akutpsychotische Entwicklung als Reaktion auf die Therapieunterbrechung gedeutet werden. Aggressive Gefühle der Patientin infolge der Therapiepause waren abgespalten und auf Angehörige projiziert worden. Kritik zu äußern, etwa an der bevorstehenden Sommerpause, war der Patientin basierend auf dieser Einsicht nun möglich. Auch andere Teilnehmer konnten über die durch die Therapieunterbrechung aktualisierten Vernichtungsängste, aggressiven Impulse und Fantasien sprechen. Die Übertragungs-Gegenübertragungsanalyse ermöglichte der Gruppenleiterin die Deutung, dass die Teilnehmer von ihr Sicherheit und die Garantie erwarteten, dass es keine Therapieunterbrechungen mehr gebe, eine Sicherheit, die sie in der Kindheit von den Eltern gebraucht hätten. Dies sei in Hinblick auf die Realität nicht möglich, aber sie könnten nun über die dadurch ausgelösten Gefühle in der Gruppe und mit der Gruppenleiterin sprechen.

Da diese Gruppe bereits seit Jahren durch den Therapieprozess eine höhere Kohäsion [33–35] und Konstanz ihrer Mitglieder aufwies, war die Gruppe als inneres gutes Objekt repräsentiert. Damit und durch die emphatische Deutungsfähigkeit der Therapeutin in der Krisensituation ging eine unmittelbare Verbesserung der Reflexionsfähigkeit der Gruppenteilnehmer einher; Therapieabbrüche oder schwerere Verläufe konnten so verhindert werden.

Im ambulanten Setting wird die Mentalisierungsfähigkeit durch ein stabiles konsistentes Setting, das Etablieren von Reflexionsräumen, wie sie durch Supervision und Intervision gegeben sind, aber auch in der Dokumentation des Therapieprozesses nach jeder Sitzung, einer Analyse der Gegenübertragung sowie der phänomenologischen Erfassung des sich im interpersonellen Raum Zeigenden erhalten (vgl. Triangulierungsfunktion).

Darüber hinaus erscheint im niedergelassenen Bereich, wo viele Kolleginnen und Kollegen in Wahlarztpraxen alleine arbeiten, die Vernetzung wichtig, die neben Supervision und Intervision spontan genutzt werden kann, wenn die Belastung eines Arbeitstages (emotional und reflexiv) stark ist. Diese psychohygienische Entlastungsmöglichkeit bedeutet eine Stärkung der reflexiven Kompetenz und damit der Mentalisierungsfähigkeit.

## Ergänzende Bemerkun﻿g﻿e﻿n zum Praxisalltag im niedergelassenen Bereich

Tage in der niedergelassenen Praxis variieren bzgl. Schwierigkeit und Komplexität der zu therapierenden Patienten. Dies bedeutet, dass Flexibilität gefragt ist, die Fähigkeit auch in unvorhergesehenen Situationen adäquat zu reagieren und die Mentalisierungsfähigkeit zu bewahren. Krisenhafte Entwicklungen (z. B. manische oder psychotische Schübe) kündigen sich nicht immer verlässlich in den Stunden zuvor an. Selbst- oder Fremdgefährdung und suizidale Einengungen halten sich nicht an den Stundenplan. Allein diese zu erkennen, verlangt Expertise. Es kann notwendig sein, die Sprechstunde zu unterbrechen und in der Situation sicherzustellen, dass die Akutversorgung gewährleistet ist und der Patient, dessen Therapiestunde unterbrochen werden muss, damit adäquat umgehen kann. In Langzeittherapien entsteht eine Beziehung, welche das Reagieren auf akute Verschlechterungen einfacher machen kann. Kompensationsstrategien und Handlungsspielraum zur Steigerung der Selbsteffizienz des Patienten konnten aufgebaut und in der Therapiestunde aktualisiert und genutzt werden. Für Therapeuten im niedergelassenen/ambulanten Setting zählt neben psychotherapeutischen Kompetenzen und Organisationstalent auch die Fähigkeit, in situativ problematischen Fragestellungen und verlangenden Tagen für eine Aufarbeitung der Belastungsmomente zu sorgen. Neben der kognitiven Reflexion ist an besonders verlangenden Praxistagen die emotionale Entlastung von Bedeutung.

## Fazit für die Praxis

- Sozialisation bedeutet Identifikation mit einer sozialen Gruppe.
- Wird diese als „gutes“ inneres Objekt internalisiert, bedeutet dies eine Stärkung der Mentalisierungsfähigkeit.
